# Detection of _OXA‐23_ Among Carbapenem‐Resistant *Acinetobacter baumannii* Isolated From Intensive Care Units of a Tertiary Care Hospital in Pakistan

**DOI:** 10.1002/mbo3.70364

**Published:** 2026-07-05

**Authors:** Hafsa Waseem, Ihsan Ullah, Shahzad Ahmad, Irfan Ali Mirza, Benish Aleem, Shazia Iqbal

**Affiliations:** ^1^ Institute of Pathology and Diagnostic Medicine (IPDM), Khyber Medical University Peshawar Pakistan; ^2^ School of Medicine, The University of Buckingham Buckingham UK; ^3^ Armed Forces Postgraduate Medical Institute (AFPGMI) Rawalpindi Pakistan

**Keywords:** blaOXA‐23, carbapenemase, carbapenem‐resistant *Acinetobacter baumannii* (CRAB), intensive care units (ICU), polymerase chain reaction (PCR)

## Abstract

Carbapenem‐resistant *Acinetobacter baumannii* (CRAB) has been designated a top‐priority critical pathogen by the World Health Organization, and the CDC has also classified it as a serious and immediate public health threat. The Cross‐sectional study was conducted to detect the *_bla_*_OXA‐23_ gene among carbapenem‐resistant *Acinetobacter baumannii* isolated from clinical specimens in intensive care units. The study was conducted at the Department of Microbiology, Institute of Pathology and Diagnostic Medicine, Khyber Medical University, from January 2024 to December 2024. Phenotypically confirmed Carbapenem‐resistant *Acinetobacter baumannii* isolates were genotypically verified via _*bla*OXA‐51_ detection, then screened for _*bla*OXA‐23_ using polymerase chain reaction, and PCR mapping was done for the upstream insertion of *ISAba1*. _*bla*OXA‐23_ gene with an upstream insertion of *ISAba1* is detected in all 52 (100%) isolates. ST19 is the most commonly identified ST, accounting for 31 (59.6%), followed by ST2 14 (26.9%) and ST1 7 (13.4%). Consequently, these findings emphasize the implementation of targeted infection control measures and routine surveillance screening within high‐risk ICU environments to mitigate further transmission.

## Graphical abstract

Graphical Abstract of the Experimental Workflow for CRAB Isolate Characterization.

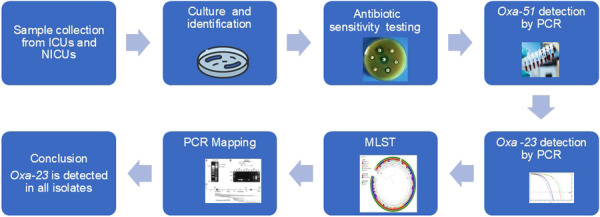

## 1 Introduction

*Acinetobacter baumannii* has emerged as a formidable nosocomial pathogen, particularly in intensive care units, notorious for its ability to acquire resistance to multiple antibiotics, including carbapenems (Kou et al. 2024; Potron et al. 2024). The rise of carbapenem‐resistant *Acinetobacter baumannii* (CRAB) poses a significant global health threat, with the World Health Organization designating it as a critical priority pathogen for the development of new antibiotics (Lu et al. 2025). Among its arsenal of resistance genes, the _blaOXA‐23_ gene, in particular, has been widely reported as a dominant carbapenemase gene contributing to resistance in CRAB isolates worldwide. and clinically important contributors to carbapenem resistance. This gene encodes a class D carbapenem‐hydrolyzing β‐lactamase, which effectively inactivates carbapenem antibiotics, often considered the last line of defense against multidrug‐resistant bacterial infections. The _*bla*OXA‐23_ enzyme enables *Acinetobacter baumannii* to survive even aggressive antimicrobial treatments, leading to limited therapeutic options and higher mortality rates (Adeyemi et al. 2025; Kim et al. 2024).

Rapid and accurate detection of _*bla*OXA‐23_ is essential for effective infection control and antimicrobial stewardship (Lari et al. 2019). Polymerase chain reaction (PCR) has become a cornerstone molecular technique for identifying resistance genes due to its high sensitivity, specificity, and rapid turnaround time (Dzugasová et al. 2022). Early detection through PCR facilitates timely intervention, reducing the spread of resistant strains and improving patient outcomes. Recent advancements, including multiplex PCR and droplet digital PCR (ddPCR), have further enhanced the detection of _*bla*OXA‐23_ in various clinical samples (Kou et al. 2024; Lellouche et al. 2024; Karampatakis et al. 2025). As antibiotic resistance continues to rise, targeted research into resistance genes, such as _*bla*OXA‐23,_ is vital to inform public health responses and guide antimicrobial stewardship efforts (Zhang et al. 2025).

The objective of the study was to detect the _*bla*OXA‐23_ gene among carbapenem‐resistant *Acinetobacter baumannii* isolated from clinical samples in intensive care units.

## 2 Materials and Methods

This study was a cross‐sectional investigation conducted at the Department of Microbiology, Khyber Medical University, following ethical approval from the Institutional Ethical Committee (Ref. No. KMU/IPDM/IEC/2023/36) on December 18, 2023. The study was carried out from January 2024 to December 2024. The sample size was calculated using the formula for descriptive studies (Charan and Biswas 2013).

Formula:

$$n=z^{2}\text{pq}/e^{2},$$

where *n* is the sample size, *z* is the standard normal variate (value is 1.96 at a 5%margin of error), *p* is the expected proportion in the population based on previous studies (Said et al. 2021) (3.5% overall average prevalence, *q* is equal to 1‐p, and e is the margin of error (0.05). The estimated sample size calculated was 52. Due to the limited availability of isolates, a non‐probability consecutive sampling approach was adopted, including all eligible CRAB isolates collected during the study period. Carbapenem‐resistant *Acinetobacter baumannii* isolated from different clinical specimens were included in the study after written informed consent was obtained.

Duplicate isolates from the same patient and *carbapenem‐sensitive Acinetobacter baumannii* were excluded from the study. All the isolates were collected from the Armed Forces Institute of Pathology (AFIP) from different clinical samples, including pus, blood, sputum, tissue, fluid, NBL, BAL, and urine. All the samples included were from the hospital's intensive care units. Isolates were identified by colony morphology, Gram staining, motility test, and 20NE to identify up to genus and species level, and were preliminarily identified as *Acinetobacter baumannii*

Carbapenem testing of all initially identified *Acinetobacter baumannii* isolates was performed using Kirby‐Bauer disk diffusion and broth microdilution, and the results were interpreted in accordance with the Clinical and Laboratory Standards Institute (CLSI) 2024 guidelines. Bacterial genomic DNA was extracted using the Bacterial XpressTM Nucleic acid extraction kit (EMD Millipore Corporation, CA92590, USA) according to the manufacturer's instructions. Genomic identification of *Acinetobacter baumannii* was done by the detection of the _*bla*OXA‐51_ gene by using the following primers:

**blaOXA‐51‐F, TAATGCTTTGATCGGCCTTG,**

**blaOXA‐51‐R, TGGATTGCACTTCATCTTGG**

52 samples confirmed to be *Acinetobacter baumannii* were included for further investigation. Polymerase chain reaction was performed for amplification of _*bla*OXA‐23_ by using the following primers: **OXA‐23‐F GATGTGTCATAGTATTCGTCG**

**OXA‐23‐R TCACAACAACTAAAAGCACTG.** The Amplification of the _*bla*OXA‐23_ gene initiated with a denaturation step at 94°C for 5 min., 30 cycles of 94°C for 25 s, 55°C for 40 s, and 72°C for 50 s, and a final elongation at 72°C for 6 min as described by Hassan RM (Hassan et al. 2021).

MLST genotyping was conducted on 52 samples by sequencing gene loci (cpn60‐fusA‐gltA‐pyrG‐recA‐rplB‐rpoB) as described by Bartual (Bartual et al. 2005). The Pasteur scheme was employed for MLST analysis*. ISAba1* forward primer and _*bla*OXA‐23_ reverse primer were used for PCR mapping.**ISAba1‐F CCTTGAATGGAGTGTATTGC**

**OXA‐23‐R TCACAACAACTAAAAGCACTG**

Initially denaturation at 95°C for 5 min, 35 cycles of 95°C for 45 s, 56°C for 45 s, and 72°C for 3 min, and a final elongation at 72°C for 5 min, as described by Turton (Turton et al. 2006).

## 3 Results

**Figure 1:**
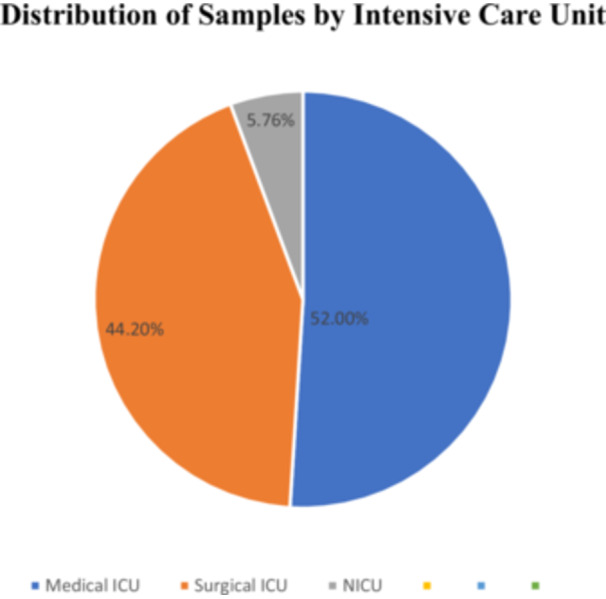
Samples collected from different intensive care units of a hospital.

**Figure 2:**
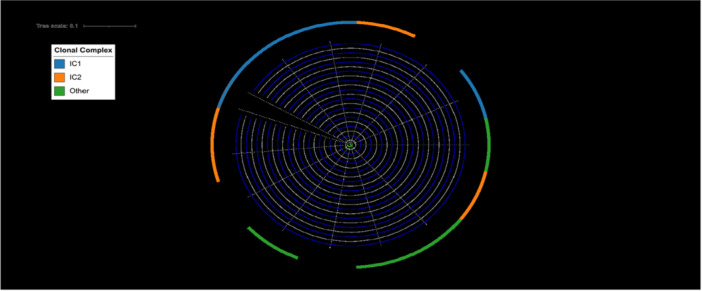
Lineage of sequence types (STs) identified.

_*bla*OXA‐51_ was detected in 52 of the phenotypically confirmed *Acinetobacter baumannii*. So, these 52 confirmed samples were included in the study. The Maximum age was 83 years, and the minimum age was 1 day. The mean age of patients with CRAB was 42.8 years (SD = 19.9). Samples from the medical ICU were 26 (52%), from the surgical ICU were 23 (44.2%), and from the NICU were 3 (5.76%), as shown in Figure 1. Most carbapenem‐resistant *Acinetobacter baumannii* isolates were obtained from pus and BAL samples, followed by tissue, blood, sputum, fluid, and urine, as shown in Table 1. Antimicrobial susceptibility pattern of Carbapenem‐resistant *Acinetobacter baumannii* is shown in Table 2. The _*bla*OXA‐23_ was detected in all CRAB isolates analyzed (52/52, 100%). Of 52 CRAB isolates, MLST analysis identified three sequence types. ST19 is the most commonly identified ST, accounting for 31 (59.6%). It belongs to Clonal complex 1 (CC1), a variant within the IC1 lineage. ST 2 is identified in 14(26.9%) isolates, which belong to Clonal Complex 2 (CC2) and correspond to international clone 2 (IC2). It is the most widespread and dominant CRAB clone globally. and ST1, with a count of 7(13.4%); it is the central genotype of Clonal Complex 1 (CC1) and corresponds to international clone 1 (IC1) as shown in Figure 2. The most common resistance gene across all three STs is _*bla*OXA‐23_. PCR mapping showed that *ISAba1* was inserted upstream of _*bla*OXA‐23_ in all 52 (100%) isolates.

**Table 1:** Samples yielded growth of CRAB.

Sample	*n* (%)
Pus	14 (26.9)
BAL	14 (26.9)
Tissue	11 (21.1)
blood	5 (9.6)
Sputum	3 (5.7)
Fluid	3 (5.7)
Urine	2 (3.8)

**Table 2:** Antibiotic susceptibility profile of 52 Carbapenem‐resistant *Acinetobacter baumannii* (CRAB).

Antibiotics	Sensitive *n* (%)	Resistant *n* (%)
Cefiderocol	52 (100)	0
Colistin	52 (100)	0
Tigecycline	35 (67.3)	17 (32.7)
Minocycline	47 (90.3)	5 (9.7)
Doxycycline	26 (50)	26 (50)
Levofloxacin	1 (2)	51 (98)
Ciprofloxacin	0	52 (100)
Gentamicin	4 (7.7)	48 (92.3)
Amikacin	0	52 (100)
Ampicillin‐Sulbactam	0	52 (100)
Tazo‐piperacillin	1 (2)	51 (98)
Ceftazidime	2 (3.8)	50 (96.2)
Cefepime	3 (5.8)	49 (94.2)
Piperacillin	0	52 (100)

## 4 Discussion

Carbapenem‐resistant *Acinetobacter baumannii* (CRAB) has emerged in recent years as a significant global health challenge, posing an alarming threat to infection control measures in nosocomial settings. It predominantly affects immunocompromised patients and those receiving mechanical ventilation, contributing to high morbidity and mortality in critical care environments (Jiang et al. 2022). The ability of *Acinetobacter baumannii* to withstand drying and harsh environmental conditions enables it to persist on non‐biological surfaces, enhancing its potential for transmission within healthcare facilities and contributing to recurrent outbreaks and sustained endemic presence in clinical settings (Müller et al. 2023)

The emergence of OXA‐type carbapenemases in clinical settings has created a critical resistance mechanism that compromises the therapeutic effectiveness of carbapenems. These enzymes play a central role in driving clonal outbreaks within intensive care units, where infections are often difficult to treat and are associated with heightened morbidity and mortality (Paneri et al. 2023).

Class D carbapenemases, including both intrinsic enzymes such as _blaOXA‐51_ and acquired ones such as _blaOXA‐23_, are the most widespread resistance determinants globally among CRAB. A recent analysis conducted as part of the Study Network of Acinetobacter as a Carbapenem‐Resistant Pathogen (SNAP) demonstrated a clear predominance of _*bla*OXA‐23_, followed by other class D carbapenemase genes (Sharma et al. 2023).

In our study, the _*bla*OXA‐23_ gene was detected in 100% of the included CRAB isolates, indicating its dominant presence in this sampled population and its role in mediating resistance. These findings are consistent with previous molecular epidemiological studies on CRAB. For instance, a study conducted across three tertiary care hospitals in collaboration with the Lahore Diagnostic Center reported the presence of the _*bla*OXA‐23_ gene in 94.8% of isolates (Arif et al. 2024). Similarly, another investigation in Lahore, involving clinical specimens collected from five major tertiary care hospitals serving a large patient population, identified the _*bla*OXA‐23_ gene in 99% of CRAB isolates (Khurshid et al. 2020).

Comparable findings have been reported in other Asian countries, highlighting the widespread prevalence of the _*bla*OXA‐23_ gene among CRAB isolates. In India, a study conducted at a 2,000‐bed tertiary care hospital reported a 94% prevalence of the _blaOXA‐23_ gene (Sharma et al. 2023). In comparison, a systematic review from the same country documented its presence in 92.6% of CRAB isolates (Paneri et al. 2023). The Antimicrobial Testing Leadership and Surveillance (ATLAS) program, which surveyed CRAB isolates across 13 countries in the Asia‐Pacific region, reported a 94.7% prevalence of the gene (Lee et al. 2023). Similarly, a recent study in South Korea found the _blaOXA‐23_ gene in 100% of CRAB isolates collected from 16 regions (Bae and Hong 2024). In the Middle East, a study conducted in major hospitals across Jordan found a 96.7% prevalence of _*bla*OXA‐23_ in isolates from intensive care unit patients (Ababneh et al. 2025). Likewise, a 2023 study from Alexandria, Egypt, reported a 94% prevalence of the _*bla*OXA‐23_ gene among carbapenemase‐producing *Acinetobacter baumannii* isolates (Sánchez‐Urtaza et al. 2023).

In contrast to our findings, the prevalence of the _*bla*OXA‐23_ gene appears to be comparatively lower in European countries and the United States. According to data from the SENTRY Antimicrobial Surveillance Program (2020–2021), the prevalence of the _*bla*OXA‐23_ gene was 80.5% in European countries and 52.2% in the United States (Castanheira et al. 2023). A global multicenter study comprising 47 countries across five world regions provided an updated perspective on the distribution of carbapenemase‐producing genes, revealing the presence of the _*bla*OXA‐23_ gene in 78% of CRAB isolates (Müller et al. 2023). In Iran, a study conducted in intensive care units of educational hospitals in Hamadan reported an 84% prevalence of _*bla*OXA‐23_ (Kafshnouchi et al. 2022). In Southeast Asia, a study from Thailand, based on isolates collected from 11 tertiary care hospitals, found the gene in 68% of CRAB isolates (Ejaz et al. 2021). Additionally, a study from Pakistan identified _blaOXA‐23_ in 49.5% of isolates and NDM‐1, a member of the metallo‐β‐lactamase (MBL) family, in 24.7% of samples (Thirapanmethee et al. 2020).

The comparatively lower prevalence of the _*bla*OXA‐23_ gene in *Acinetobacter baumannii* isolates reported from Europe, the United States, and certain other countries, compared with our study, can be attributed to multiple factors. One of the most significant reasons is the presence of well‐developed infection prevention and control protocols in high‐income countries, including active surveillance, strict aseptic techniques, and isolation of infected patients, which effectively reduce nosocomial transmission of carbapenem‐resistant strains. Additionally, comprehensive antimicrobial stewardship programs in these regions limit the overuse of broad‐spectrum antibiotics, particularly carbapenems, thereby reducing the emergence of resistance genes such as _*bla*OXA‐23_. In contrast, the frequent misuse of antibiotics in countries such as Pakistan contributes considerably to the emergence of resistant strains. Furthermore, the healthcare infrastructure in Pakistan, including overcrowded hospitals, limited ICU capacity, and weaker infection control practices, facilitates the spread of multidrug‐resistant organisms.

It is also very important to note that _*bla*OXA‐23_ prevalence rates can vary significantly due to differences in sample collection sources, sampling techniques, and laboratory detection methods.

## 5 Conclusion

This study demonstrates a 100% prevalence of the _*bla*OXA‐23_ gene with upstream *ISAba1* among the tested CRAB isolates, highlighting a high clonal prevalence of ST19 within our facility's medical and surgical ICUs. These findings underscore the critical need to consider routine PCR screening and targeted infection control strategies, specifically in high‐risk ICU environments, to curb further transmission.

## Author Contributions

**Hafsa Waseem:** conceptualization, validation, investigation, writing – original draft, writing – review and editing, formal analysis. **Ihsan Ullah:** conceptualization, methodology, supervision, project administration, validation, visualization. **Shahzad Ahmad:** conceptualization, writing – review and editing, writing – original draft, validation. **Irfan Ali Mirza:** methodology, writing – review and editing, writing – original draft, investigation, software. **Benish Aleem:** methodology, validation, writing – review and editing, conceptualization, writing – original draft. **Shazia Iqbal:** methodology, writing – review and editing, writing – original draft. All authors have read and approved the final version of the manuscript and consent to its submission for publication.

## Funding

The authors have nothing to report.

## Ethics Statement

Ethical approval was taken from the Institutional Ethical Committee (Ref. No. KMU/IPDM/IEC/2023/36), Khyber Medical University, on December 18, 2023.

## Consent

Written informed consent was taken from patients before including the samples in the study.

## Conflicts of Interest

The authors declare no conflicts of interest.

## Limitation of Study

This study utilized a non‐probability sampling strategy, which may introduce selection bias and limit generalizability. The findings reflect the molecular characteristics of isolates included and should not be interpreted as population‐level prevalence estimates.

## Data Availability

The data that support the findings are available on request from the corresponding authors.
